# The Similarity Degree of the Anthropometric and Body Composition Variables of Brazilian and Mozambican Schoolchildren: A New Approach Using the Smoothed Jaccard Index Surface

**DOI:** 10.3390/children11070804

**Published:** 2024-06-30

**Authors:** André Luiz de Góes Pacheco, Willian Motta Bocanegra, Élida Karina de Lira Ferreira, Rayssa Temudo dos Santos, Patrícia Miller Simões, Eulálio Malinga, Euclides da Conceição Guiliche, Arsénio Fernando José Isaías, Isabele Góes Nobre, João Henrique da Costa Silva, Wylla Tatiana Ferreira e Silva, Eduardo Padrón-Hernandez, Luciano Pirola, Rafael dos Santos Henrique, Carol Góis Leandro

**Affiliations:** 1 Center of Informatic, Universidade Federal de Pernambuco, Recife 50670-901, Brazil; algp@cin.ufpe.br (A.L.d.G.P.); willian.bocanegra@ufpe.br (W.M.B.); elida.lira@ufpe.br (É.K.d.L.F.); 2 Department of Nutrition, Centro Acadêmico de Vitória (CAV), Universidade Federal de Pernambuco, Recife 50670-901, Brazil; rayssa.temudo@ufpe.br (R.T.d.S.); patricia.millersimoes@ufpe.br (P.M.S.); isabele.gnobre@ufpe.br (I.G.N.); wylla.silva@ufpe.br (W.T.F.e.S.); 3 Faculty of Physical Education and Sports, Universidade Pedagógica de Maputo, Maputo 1100, Mozambique; eulalio.malinga@ufpe.br (E.M.); euclides.guiliche@ufpe.br (E.d.C.G.); aisaias@unisave.ac.mz (A.F.J.I.); 4 Department of Physical Education and Sports Science, Centro Acadêmico de Vitória (CAV), Universidade Federal de Pernambuco, Recife 50670-901, Brazil; joao.hcsilva@ufpe.br; 5 Department of Physics, Universidade Federal de Pernambuco, Recife 50670-901, Brazil; eduardo.hernandez@ufpe.br; 6 CarMeN (Cardiology, Metabolism and Nutrition) Laboratory, INSERM U1060, South Lyon Medical Faculty, Lyon-1 University, 69921 Oullins, France; luciano.pirola@univ-lyon1.fr; 7 Department of Physical Education, Universidade Federal de Pernambuco, Recife 50670-901, Brazil; rafael.shenrique@ufpe.br

**Keywords:** growth, pediatric healthcare, childhood, nutritional status, Mozambique, Brazil

## Abstract

Background/Objectives: Brazil and Mozambique face similar socioeconomic challenges, including common indicators of undernutrition and overnutrition among children. This study evaluated the similarity degree of the anthropometric and body composition variables of Brazilian and Mozambican children by using the Jaccard index. Methods: A total of 1831 children of both genders aged 7–10 years from three Brazilian cities (Recife, Vitoria de Santo Antao, and Lagoa do Carro) and three Mozambican cities (Maputo, Boane, and Inhambane) participated in this study. Anthropometric (height, body mass, and waist circumference) and body composition (body fat percentage [%BF], lean mass, and fat mass) variables were measured and the Smoothed Jaccard Index Surface (SJIS) was used to evaluate the similarity degree. Results: Brazilian children were taller and heavier and had a higher %BF and fat mass than Mozambican children. Children living in urban areas were taller than those living in rural zones in both countries. Brazilian and Mozambican children showed high similarity only between %BF and lean mass. Children from Recife and Maputo had high similarities among waist circumference, body mass, fat mass, height, and %BF. Finally, a high SJIS degree was observed among height and %BF for schoolchildren from rural and urban zones. Conclusion: Brazilian and Mozambican children exhibit differences in growth characteristics but a high degree of similarity when children from rural and urban zones are compared.

## 1. Introduction

The normal growth curve reported by the World Health Organization (WHO) was derived from longitudinal studies that tracked the physical development of a large and diverse population over time [1]. These studies collected data on height, weight, head and hip circumferences, and other growth-related parameters at specific ages, allowing the creation of growth charts that healthcare professionals can use to monitor and assess individual growth [2]. Growth charts are often used in pediatric healthcare to assess a child’s development and identify potential growth-related problems [3].

It is important to note that variations in individual growth trajectories may differ in different countries and regions of the same country (i.e., rural and urban areas), even if individuals fit well into the standard growth curve [4,5,6,7,8]. A multicentric study including 10 countries showed the lowest mean values for body mass index (BMI) and height compared with the reference values proposed by the WHO, the Centers for Disease Control and Prevention (CDC), the International Obesity Task Force (IOTF), and the Dutch Growth Study (DUTCH) [9,10]. Children who experience deviations from normal growth patterns, such as being underweight or overweight, are at higher risk of developing cognitive impairment, delayed motor skill development, low self-esteem, and sedentary behavior [11,12]. In addition, children who experience rapid weight gain or obesity early in life are more likely to struggle with weight management and associated health issues throughout their lives and are more likely to develop type 2 diabetes, cardiovascular diseases, and hypertension in adulthood [12,13].

By comparing the measured values of an individual or a population with the expected values from the growth chart, healthcare providers can assess whether a child is growing within the normal range or whether further investigations and interventions are needed [3]. Early detection and intervention in growth-related issues play critical roles in promoting healthy development, preventing long-term health consequences, and addressing concerns early on to support children in reaching their full growth potential and thriving both physically and mentally [3,4]. Calculating the similarity degree between populations helps to assume that other factors, such as genetics, nutrient availability, and physical activity, may influence the normal growth trajectory according to the socioeconomic and cultural environment.

Brazil and Mozambique share several connections, despite being in different continents. Both are former Portuguese colonies, and this linguistic connection influences culture, gastronomy, society, and institutional exchange. Both countries are also rich in natural resources, including minerals, agriculture, and extensive coastlines. In addition, both Brazil and Mozambique face similar socioeconomic challenges, including poverty, social inequality, a low availability of healthcare services, education, and infrastructure development. Social inequality between rural and urban zones in both countries has deep historical roots and persist, despite efforts to address it [11].

Brazil and Mozambique share common indicators of malnutrition, including a high prevalence of both undernutrition (i.e., stunting, wasting, and underweight) and overnutrition (i.e., obesity and overweight) among children and adolescents [12,14]. In addition, there are differences between rural and urban areas in both countries, as the availability of food is unequally distributed in both countries [13,15]. However, less is known about the degree of similarity in terms of anthropometric and body composition variables among multiethnic populations. The Jaccard similarity coefficient (or Jaccard index) is a measure often used to compare and evaluate the similarity between two datasets and determine how much overlap is possible. Sets of patterns were compared using the Jaccard index in previous studies [16,17,18].

The present study evaluated the similarity degree of the anthropometric and body composition variables of children aged 7–10 years living in either Brazil or Mozambique, as well as between rural and urban zones, using the Jaccard index. The findings of this study establish robust monitoring and evaluation mechanisms to track progress in reducing infant undernutrition and overweight in low-middle income countries. In addition, it can be used for public health policies and interventions, ensuring that children receive appropriate nutritional support and primary healthcare services to support healthy growth trajectories.

## 2. Materials and Methods

### 2.1. Sample Populations

This study utilized data from the project “Growing Healthy”, representative research conducted in the years 2019–2023 with schoolchildren of both genders living in either rural or urban zones in northeast Brazil (Recife, Vitoria de Santo Antão, and Lagoa do Carro) and Mozambique (Maputo, Boane, and Inhambane). This study was conducted under the leadership of the Universidade Federal de Pernambuco in Brazil.

Children aged 7–10 years were considered eligible for this study, of which 1831 individuals of both genders (907 girls and 924 boys) participated in the study (Table 1). The Brazilian children came from three different cities: (1) Recife, the capital of Pernambuco [273 schoolchildren (114 girls and 159 boys)]; (2) Vitória de Santo Antao, located in the rural zone of the Pernambuco state [352 children (174 girls and 178 boys)]; and (3) Lagoa do Carro, a city located in a rural zone of Pernambuco [663 children (331 girls and 332 boys)].

In total, 543 Mozambican schoolchildren aged 7 to 10 years from three different cities were evaluated: (1) Maputo, the capital of Mozambique [147 children (71 girls and 74 boys)]; (2) Boane, a city in a rural zone of Mozambique [220 children (122 girls and 98 boys)]; and (3) Inhambane Province, located in the southern rural region of Mozambique [176 children (95 girls and 81 boys)] (Table 1).

Children with chronic illnesses and neuromotor disability were excluded. Only children from public schools were chosen to participate, and they did not have differences in socioeconomic characteristics. Written informed consent was obtained from the parents or legal guardians of each child.

### 2.2. Ethical Procedures

All studies were conducted according to the Declaration of Helsinki. In Mozambique, the Ethics Committee of the National Bioethics Committee for Health of Mozambique approved the procedures of the study in all three cities (Maputo: Ref. 828/CNBS/22). Data were collected from 22 January to 18 December 2023. The Research Ethics Committee of the Federal University of Pernambuco approved the studies in Vitória de Santo Antão (Protocol: 5.159.516, data were collected from 2 February 2022 to 19 August 2023) and Recife (Protocol: 1.853.815, data were collected from 5 February to 14 December 2019). Lagoa do Carro’s study was approved by the Ethics and Research Committee of the University of Pernambuco (Protocol: 2.520.417, data were collected from 3 February to 1 December 2019). Written informed consent was obtained from the parents or legal guardians of each child and in each study. The authors had no access to information that could identify individual participants during or after data collection.

### 2.3. Anthropometry and Body Composition

Height was measured to the nearest 0.1 cm using a portable stadiometer (Holtain, Crymych, UK) with the participant’s head positioned in the Frankfurt horizontal plane [19]. Body mass was measured to the nearest 0.1 kg using a digital scale (Secca, Hamburg, Germany). The triceps and subscapular skinfolds were measured using a Langer caliper (Lange, Santa Cruz, CA, USA). Waist circumference was measured using a non-elastic anthropometric tape (Sanny, São Paulo, Brazil) to the nearest 0.1 cm. Body fat percentage, fat mass (kg), and lean mass (kg) were calculated using previously described formulas [20]. All measurements were performed according to the procedures outlined in a previous study [21].

### 2.4. Statistical Analysis

Initially, an exploratory analysis was performed to identify possible tabulation errors or the presence of outliers. Descriptive statistics are presented as means, standard deviations, and minimum and maximum values. Normality was assessed using the Kolmogorov–Smirnov test, whereas homogeneity of variance was assessed using the Levene test. One-way ANOVA was used to test for differences between cities, between rural and urban areas, and between Brazilian and Mozambican children. Bootstrapping procedures were performed (1000 resamples; 95% CI BCa) to obtain greater reliability of the results and to correct for deviations from the normality of the sample distribution and differences between group sizes [22] with the Games–Howell post hoc test when necessary [22]. Effect sizes were assessed (for pairwise comparisons) using standardized mean differences, considering ≤ 0.2 very small, 0.2–0.5 small, 0.6–0.8 moderate, and >0.8 large differences [22]. These analyses were performed using SPSS 23 (IBM SPSS Corporation, New York, NY, USA).

The Jaccard index is a key metric for analyzing set similarity across various sets and plays a vital role in quantifying the similarity between vertex neighbor sets in graphs. A graph, denoted as *G* = (*V*,*E*), consists of a vertex set *V* and edge set *E*, where each edge represents a connection between two vertices. The neighbor set of a vertex *v* denoted *N*(*v*) comprises all vertices directly connected to *v*. The Jaccard index for vertices *i* and *j* in graphs *G*_1_ and *G*_2_ is calculated as the ratio of the intersection to the union of their neighbor sets, expressed mathematically as follows:

(1) $$J\left(i,j\right)=\frac{\left|N_{G_{1}}(i)\cap N_{G_{2}}(i)\right|}{\left|N_{G_{1}}(i)\cup N_{G_{2}}(i)\right|}$$

Here, *N_G_*_1_ (*i*) ∩ *N_G_*_2_ (*j*) represents the size of the intersection of the neighboring sets of vertex *i* in graph *G_1_* and vertex *j* in graph *G*_2_. This intersection includes all vertices that are neighbors of both *i* in *G*_1_ and *j* in *G*_2_, and ∣*N_G_*_1_ (*i*) ∪ *N_G_*_2_ (*j*)∣ represents the size of the union of the neighboring sets of the vertex. This formula measures the similarity of the local environments of vertices *i* and *j* in their respective graphs, ranging from 0 (no similarity) to 1 (complete similarity) [18]. Peaks on the surface represent areas of high similarity, whereas valleys represent lower similarities, indicating the presence of a greater influence of other environmental characteristics on the similarity of such variables. A high degree of similarity is considered when values greater than 0.7 are observed [18]. These analyses were performed using pandas, numpy, network, matplotlib, and griddata [18].

## 3. Results

Descriptive statistics for the anthropometric and body composition data are presented in Table 1. All anthropometric and body composition characteristics differed between Mozambique and Brazil. In terms of body height, children from Maputo and Inhambane were shorter than Brazilian children (Recife, Vitória de Santo Antão, and Lagoa do Carro; all *p*-values were <0.001), while children from Boane were shorter than those from Recife and Lagoa do Carro (*p* < 0.05).

Children from Recife, the capital of Pernambuco, were taller than those from the rural zone of the same state (Vitória de Santo Antão and Lagoa do Carro). Brazilian children also had higher body weights than children from all the cities in Mozambique (Maputo, Boane, and Inhambane), although children from Recife were heavier than those from the rural zone (i.e., Vitória de Santo Antão and Lagoa do Carro). Children from Maputo, the capital of Mozambique, had lower values than those from all other cities in Brazil and Mozambique (all *p*-values < 0.01). On the other hand, children from Maputo had lower waist circumferences than those from Recife, Vitoria de Santo Antão, and Lagoa do Carro (Table 1).

Regarding body composition, children from all Brazilian cities had higher fat percentages and fat mass values than those from Mozambican cities. However, no significant differences were observed between cities in the same country (*p* > 0.05). When considering lean mass, children from both capitals showed a significant difference compared to the other cities; those from Maputo had a lower lean mass than those from all the other cities (all with *p* < 0.05), while those from Recife had a higher lean mass than those from other cities (Table 1).

Brazilian children showed higher values than Mozambican children for all anthropometric and body composition characteristics (Table 2). The differences ranged from low (height and lean mass; effect sizes = 0.45 and 0.32, respectively) to high (fat percentage; effect size = 1.06). When comparing children from urban and rural areas, differences with very low (0.06) to low effect sizes (0.24) were observed in all characteristics, except height, where no significant differences were observed (Table 2).

**Figure 1:**
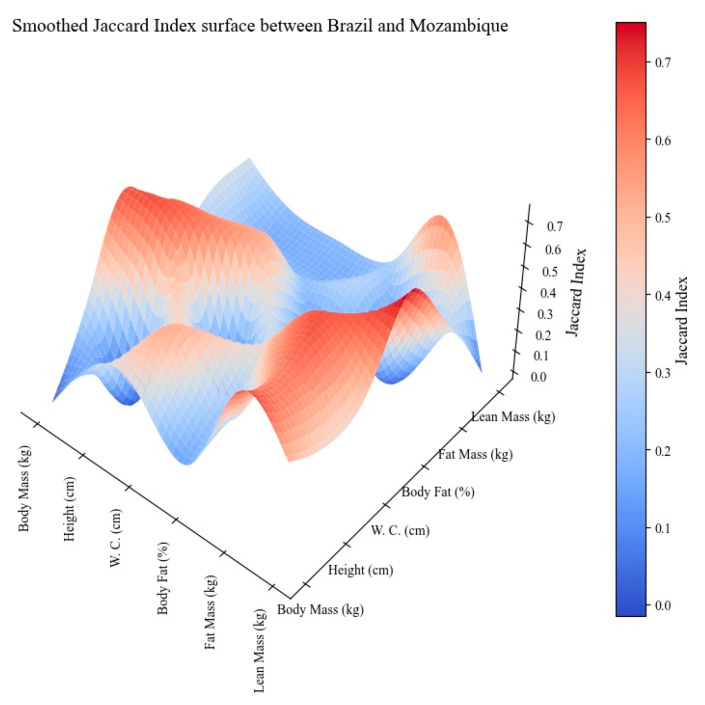
Smoothed Jaccard Index Surface between Brazil and Mozambique.

Figure 1 shows the Smoothed Jaccard Index Surface between Brazil and Mozambique. Most comparisons showed low similarity (≤0.5), particularly in the relationships between body mass and %BF, height and lean mass, waist circumference and lean mass. We also observed moderate similarities (between 0.5 and 0.7) between waist circumference and body mass, waist circumference and %BF, and waist circumference and fat mass (with indices around 0.6 and 0.67, respectively). These moderate similarities may indicate some consistency in the relationships between body fat distribution and body mass among the countries. A high similarity was noted only between the %BF and lean mass (0.75).

**Figure 2:**
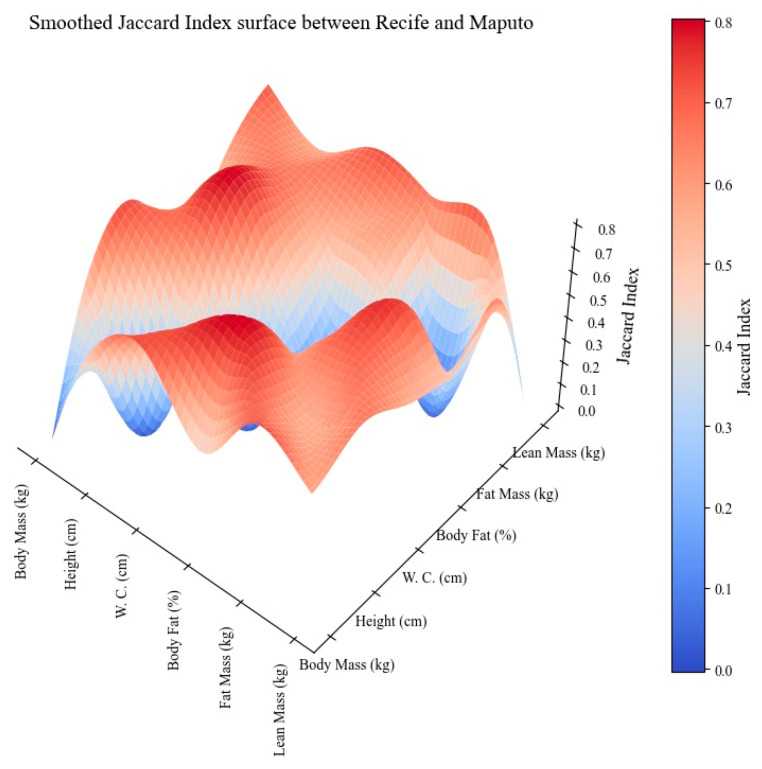
Smoothed Jaccard Index Surface between Recife and Maputo.

Figure 2 shows the Smoothed Jaccard Index Surface between Recife and Maputo. The only low similarity observed was between %BF and body mass (0.42). Several characteristics showed moderate similarities, notably, height and lean mass, both with indices of 0.57, and height with %BF with indices of 0.67. High similarities are more prevalent and include relationships such as waist circumference with body mass and fat mass, both of which have indices of 0.71, and height with %BF, with an index of 0.8. Height and lean mass, as well as height and %BF, showed moderate similarity at 0.57 and 0.67, respectively. High similarities were observed between waist circumference with body mass and fat mass, both with indices of 0.71, and height with %BF, with an index of 0.8.

**Figure 3:**
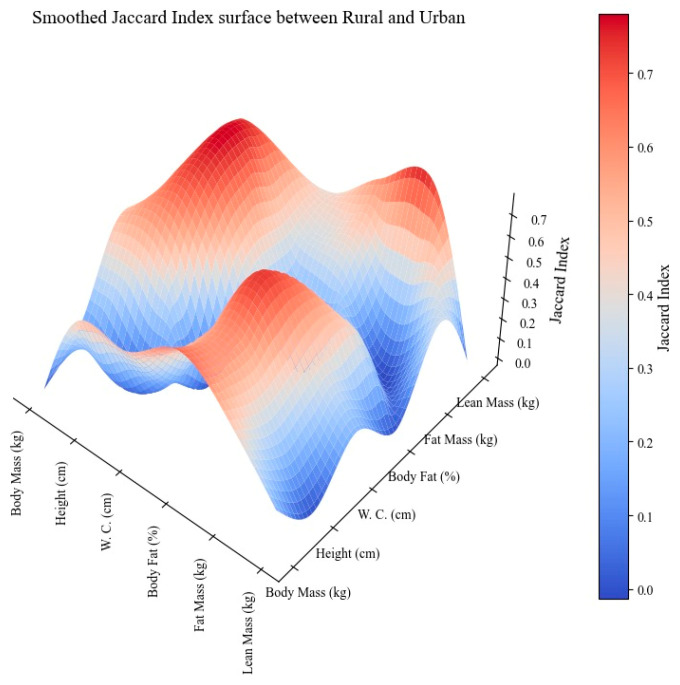
Smoothed Jaccard Index Surface between rural and urban zones.

Finally, the Smoothed Jaccard Index Surface between rural and urban zones is depicted in Figure 3. Most comparisons between rural and urban zones showed low similarity, especially between body mass and lean mass, height and lean mass, and %BF. Moderate similarity was found in several comparisons, such as between body mass and height, body mass and waist circumference, and waist circumference and %BF, with indices ranging from 0.5 to 0.67. A high similarity was observed in the comparison between height and %BF, with an index of 0.75.

## 4. Discussion

In Brazil, several public policies aimed at reducing infant undernutrition have been implemented over the past few decades and have contributed to significant improvements in child nutrition outcomes across the country [23,24,25]. Examples include the Zero Hunger Program, National School Feeding Program (PNAE), National Policy for Food and Nutrition Security (PNSAN), and Nutritional Guidelines for Children [23,24,25].

Similarly, Mozambique has implemented various public policies and initiatives aimed at reducing infant undernutrition and improving child health outcomes. For example, Mozambique’s National Food and Nutrition Security Strategy (ENSAN) addresses the root causes of food insecurity and malnutrition. The strategy focuses on improving access to nutritious food, promoting breastfeeding and complementary feeding practices, enhancing agricultural productivity, and strengthening social safety nets for vulnerable populations [13,26,27]. Infant populations reflect broader socioeconomic conditions, including poverty levels, education levels, employment opportunities, and social inequalities. Comparing these factors between Brazil and Mozambique can highlight the structural determinants of health disparities and inform policies aimed at reducing poverty and promoting social equity.

Comparing pediatric populations in Brazil and Mozambique can provide valuable insights into various aspects of public health, healthcare systems, socioeconomic conditions, and developmental outcomes in these two countries. For examples, understanding how these factors interact with health outcomes can guide interventions aimed at addressing inequalities and promoting health equity among children from diverse backgrounds. In this study, schoolchildren from Brazil were taller and heavier than those from Mozambique. According to the nutritional indices weight-for-age, height-for-age, BMI-for-age, and weight-for-height used by the WHO growth standard, 86.4% of the children from both countries were classified as normal or eutrophic (healthy range, 5th to <85th percentile), 10.2% were classified as thin (<5th percentile: severely underweight, stunted, or wasted, indicating acute malnutrition), and 3.4% were classified as overweight (85th to <95th percentile, indicating a risk and/or predisposition toward overt obesity, >95th percentile). These findings can guide targeted interventions, policy recommendations, and collaborative efforts to improve child health outcomes and address health disparities in both countries.

In this study, children from all Brazilian cities had higher %BF and fat mass values than those from Mozambican cities. Accordingly, previous studies have shown that %BF in children can vary among different countries due to a variety of factors, including genetic, environmental, cultural, dietary, and lifestyle differences [28,29,30,31]. However, in terms of lean mass, rural and urban zones differed in Brazil and Mozambique. Variations in physical activity levels among children in different countries can affect lean mass [23,32,33,34]. Previous studies considering a secular trend have shown that children from Brazil and Mozambique exhibit increased physical activity [29,35,36,37]. Countries with active lifestyles or extensive opportunities for outdoor play and sports participation may have higher levels of lean mass among children than more sedentary populations [12]. Overall, comparing infant populations in both rural and urban zones in Brazil and Mozambique offers a valuable opportunity to identify challenges, share lessons learned, and collaborate on strategies to improve maternal and child health outcomes in both countries and contribute to global efforts to reduce health inequalities.

The Smoothed Jaccard Index Surface between Brazil and Mozambique showed high similarity between %BF and lean mass (0.75), which may reflect common body composition patterns, despite cultural and environmental differences. Cultural dietary habits and food availability can significantly impact %BF and lean mass [38]. Indeed, both Brazil and Mozambique are concerned about healthy diets rich in fruits, vegetables, whole grains, and lean proteins to promote a healthier body composition, especially in children and adolescents during development [31,39].

When we used the Smoothed Jaccard Index Surface to compare Recife and Maputo, there were some moderate similarities, notably in height and lean mass, height, and %BF, suggesting that growth patterns and body compositions are not strongly aligned but still share some similarities. However, we observed a high similarity between cities for some parameters, such as waist circumference with body mass and fat mass, and height with %BF. These high similarities indicate strong correlations between these physical characteristics in the populations of Recife and Maputo, exhibiting consistent patterns of body composition and mass distribution that transcend regional differences [28,40].

Social inequality between rural and urban zones in Brazil and Mozambique has deep historical roots and persists, despite efforts to address it. There are notable differences between the rural and urban zones in both countries in terms of anthropometry and body composition variables, such as the relationships between body mass and height, body mass and waist circumference, and waist circumference and %BF, possibly due to variations in physical activity patterns and nutrition. Previous studies have shown that the growth and development in children living in rural and urban zones differ [32,41,42]. However, there was a high similarity in the comparison between height and %BF. This similarity shows a strong correlation between height and %BF in rural and urban populations, which may indicate genetic influences or similar growth patterns that transcend environmental differences [12]. Future research should focus not only on anthropometric and body composition variables but also on investigating the degree of similarity between dietary and physical activity behaviors.

This study has some potential limitations. Despite common cultural influences, Brazilian and Mozambican children have also marked differences in biological characteristics and socioeconomic and environmental contexts. These differences could have confounded the findings, making it challenging to isolate the effects of specific factors on child health outcomes. However, evaluating of the degree of similarities between countries using the Smoothed Jaccard Index enhances our findings. Despite the large sample of schoolchildren, the sample was not completely representative of 7- to 10-year-old children from all cities. However, we included children from three different cities in each country, including rural and urban zones. Assessments throughout the entire course of childhood could provide more robust data to support the implementation of public policies in the region. We acknowledge the limitation of not including children from private schools in the study. To address this limitation, future studies could consider sampling from a more diverse range of schools, including both public and private institutions, to capture a broader socioeconomic spectrum and enhance the representativeness of the study sample. Another limitation of this study is the lack of data on children’s food consumption and physical activity, which are factors that can directly influence growth characteristics. It is important to consider that the children in this study exhibited growth indicators within the normal parameters recommended by the WHO. Nevertheless, future studies could include other biological and behavioral information to help adjust the degree of similarity of these characteristics.

## 5. Conclusions

This study evaluated the similarity degree of the anthropometric and body composition variables of children aged 7–10 years living in either Brazil or Mozambique, as well as between rural and urban zones, using the Jaccard index. We found that schoolchildren from Brazil were taller and heavier and had higher %BF and fat mass than schoolchildren from Mozambique, but most of all, children were within the normal anthropometric parameters recommended by the WHO. Our findings showed that children living in urban areas were taller than those living in rural zones in both countries.

The use of the Smoothed Jaccard Index Surface between Brazil and Mozambique showed high similarity only between %BF and lean mass. However, when Recife was compared with Maputo, high similarities were seen between waist circumference and body mass and fat mass, and height and %BF. Finally, a high degree of Smoothed Jaccard Index Surface between rural and urban zones was observed in the comparison between height and %BF.

In conclusion, Brazil and Mozambique have established robust monitoring and evaluation mechanisms to track progress in reducing infant undernutrition and overweight. Several investments in expanding access to primary healthcare services, including prenatal care, child health consultations, and nutritional counseling have made Brazil and Mozambique important partners in combating infant hunger and poverty.

## Figures and Tables

**Table 1:** Descriptive data (means ± SDs, minima and maxima) of 7–10-year-old children from Mozambican and Brazilian cities.

Variables	Maputo (*n* = 220)			Boane (*n* = 147)			Inhambane (*n* = 176)		
	Mean ± SD	Min.	Max.	Mean ± SD	Min.	Max.	Mean ± SD	Min.	Max.
Mozambique									
Age (years)	8.02 ± 0.96	7.0	9.8	8.09 ± 1.06	7.3	10.0	7.99 ± 1.04	7.3	10.0
Anthropometrics									
Height (m)	129.08 ± 8.07 ^a,φ^	106.00	165.00	132.15 ± 8.37 ^b^	112.00	156.00	130.97 ± 7.23 ^a^	115.00	155.00
Body mass (kg)	23.92 ± 4.38 ^a,φ^	19.00	41.00	28.26 ± 5.05 ^a^	17.40	42.30	29.17 ± 4.53 ^a^	19.00	41.00
Waist circumference (cm)	53.30 ± 1.97 ^a,φ^	44.50	59.50	57.95 ± 3.70	51.00	71.00	57.80 ± 5.26	49.9	72.00
Body composition									
Body fat (%)	13.98 ± 3.69 ^a^	6.59	26.65	14.57 ± 4.28 ^a^	7.47	29.62	11.88 ± 4.25 ^a^	3.40	22.10
Lean mass (kg)	20.47 ± 3.37 ^a,φ^	12.66	30.33	24.04 ± 3.81	15.02	35.16	25.32 ± 2.94	18.70	33.70
Fat mass (kg)	3.50 ± 1.59 ^a^	1.25	11.59	4.22 ± 1.87 ^a^	1.66	11.52	4.53 ± 1.42 ^a^	2.00	9.60
Brazil	**Recife (*n* = 273)**			**Vitória de Santo Antão (*n* = 352)**			**Lagoa do Carro (*n* = 663)**		
Anthropometrics									
Age (years)	8.12 ± 1.0	7.5	10.0	8.29 ± 1.20	7.2	10.0	7.89 ± 0.94	7.0	10.0
Height (m)	136.66 ± 9.34 *	115.50	164.50	133.87 ± 8.78	105.00	158.20	134.67 ± 9.73	103.50	171.00
Body mass (kg)	34.78 ± 9.78 *	18.40	66.60	32.20 ± 9.06	16.90	72.00	32.69 ± 9.60	15.00	74.30
Waist circumference (cm)	61.83 ± 8.75 *	45.00	92.50	59.46 ± 9.15	44.19	108.00	60.86 ± 8.48	43.70	96.90
Body composition									
Body fat (%)	23.04 ± 10.82	7.80	65.02	22.63 ± 9.37	6.63	62.14	23.76 ± 10.87	7.27	70.90
Lean mass (kg)	25.92 ± 4.77 *	14.79	39.71	24.28 ± 4.70	11.51	48.71	24.08 ± 4.50	13.67	51.79
Fat mass (kg)	8.85 ± 6.92	1.93	40.51	7.93 ± 5.51	1.12	38.88	8.61 ± 6.70	1.33	49.48

^a^ *p* < 0.001 compared with Recife, Vitória de Santo Antao, and Lagoa do Carro. ^b^ *p* < 0.05 compared with Recife and Lagoa do Carro. ^φ^ *p* < 0.05 compared with Boane and Inhambane. * *p* < 0.05 compared with Vitória de Santo Antao and Lagoa do Carro.

**Table 2:** Descriptive data (means ± SDs, minimum and maximum values) of 7–10-year-old children from urban cities (Maputo and Recife) and urban cities (Boane, Inhambane, Vitória de Santo Antão, and Lagoa do Carro).

Variables	Urban (n = 493)			Rural (n = 1338)			Mean Difference (95% CI)	Cohen’s d
	Mean ± SD	Min.	Max.	Mean ± SD	Min.	Max.		
Anthropometrics								
Height (m)	133.24 ± 9.56	106.00	165.00	133.75 ± 9.03	103.5	171.00	−0.50 (−1.42 to 0.41)	0.06 (very small)
Body mass (kg)	29.88 ± 9.50	15.00	66.60	**31.77 ± 8.79**	15.00	74.30	−1.89 (−2.78 to −0.99)	0.21 (small)
Waist circumference (cm)	57.98 ± 7.86	64.50	92.50	**59.69 ± 8.34**	24.30	108.00	−1.70 (−2.53 to −0.88)	0.21 (small)
Body composition								
Body fat (%)	18.95 ± 9.52	6.59	65.02	**21.37 ± 10.07**	3.40	70.90	−2.42 (−3.41 to −1.43)	0.24 (small)
Lean mass (kg)	23.47 ± 4.99	12.66	39.71	**24.29 ± 4.43**	11.51	51.79	−0.82 (−1.27 to −0.36)	0.18 (very small)
Fat mass (kg)	6.44 ± 5.87	1.25	40.51	**7.55 ± 5.74**	1.12	49.48	−1.12 (−1.69 to −0.54)	0.19 (very small)

Significant values are indicated in bold (*p* < 0.01).

## Data Availability

Data are available upon request.
